# Analysis by *in Situ* Hybridization of Cells Expressing mRNA for Tumor-Necrosis Factor in the Developing Thymus of Mice

**DOI:** 10.1155/1992/48713

**Published:** 1992

**Authors:** Johanne Deman, Marie-Thérèse Martin, Philippe Delvenne, Chantal Humblet, Jacques Boniver, Marie-Paule Defresne

**Affiliations:** Laboratory of Pathological Anatomy and Cytopathology Department of Pathology University Hospital of Liège B35 Liège B-4000 Belgium

**Keywords:** *In situ* hybridization, tumor necrosis factor-*α*, thymus, ontogeny, mouse

## Abstract

We have used *in situ* hybridization to investigate the expression of TNF-*α* genes by thymic cells during fetal development in mice. In 14-day-old fetal thymuses, very scarce cells produce TNF-*α* mRNA. A second phase of cytokine gene expression starts on day 16. The density of positive cells progressively increases up to day 20. Thymuses at 15 days of gestation and after birth do not express detectable cytokine mRNA. In an attempt to identify the nature of the TNF-*α*  mRNA-producing cells, acid phosphatase activity, which is characteristic of the macrophage lineage, was studied in the same thymuses. Acid phosphatase-positive cells only appear on day 15. Their frequency increases up to birth. However, no correlation can be established between acid phosphatase—and TNF*α* mRNA— positive cells. The results indicate that a small subset of thymic cells is responsible for TNF-*α*  mRNA production during ontogeny: These cells are not yet identified. The possible role of  TNF-*α* in thymic ontogeny is discussed.


*Developmental Immunology*, 1992, Vol. 2, pp. 103–109
Reprints available directly from the publisher
Photocopying permitted by license only

© 1992 Harwood Academic Publishers GmbH
Printed in the United Kingdom

### Analysis by *In Situ* Hybridization of Cells Expressing mRNA for Tumor-Necrosis Factor in the Developing Thymus of Mice

JOHANNE DEMAN, MARIE-THÉRÈSE MARTIN, PHILIPPE DELVENNE, CHANTAL HUMBLET, JACQUES BONIVER, and MARIE-PAULE DEFRESNE*

*Laboratory of Pathological Anatomy and Cytopathology, Department of Pathology, University Hospital of Liège B35, B-4000 Liège, Belgium*

We have used *in situ* hybridization to investigate the expression of TNF-$\alpha$ genes by thymic cells during fetal development in mice. In 14-day-old fetal thymuses, very scarce cells produce TNF-$\alpha$ mRNA. A second phase of cytokine gene expression starts on day 16. The density of positive cells progressively increases up to day 20. Thymuses at 15 days of gestation and after birth do not express detectable cytokine mRNA. In an attempt to identify the nature of the TNF-$\alpha$ mRNA-producing cells, acid phosphatase activity, which is characteristic of the macrophage lineage, was studied in the same thymuses. Acid phosphatase-positive cells only appear on day 15. Their frequency increases up to birth. However, no correlation can be established between acid phosphatase—and TNF$\alpha$ mRNA—positive cells. The results indicate that a small subset of thymic cells is responsible for TNF-$\alpha$ mRNA production during ontogeny: These cells are not yet identified. The possible role of TNF-$\alpha$ in thymic ontogeny is discussed.

KEYWORDS: *In situ* hybridization, tumor necrosis factor-$\alpha$, thymus, ontogeny, mouse.

### INTRODUCTION

A central issue of T-cell ontogeny is the identification of the signals and growth factors required for the proliferation and differentiation of immature cells in the thymus. TNF-$\alpha$ may be one of these signals. This protein was initially described as cytostatic and cytotoxic against a variety of tumor cells (Carswell et al., 1975) and normal cell types (Broxmeyer et al., 1986). Besides, TNF-$\alpha$ can induce a number of negative and positive regulators of cell growth [e.g., granulocyte-macrophage CSF (Munker et al., 1986), IL-1 (Nawroth et al., 1986), IFN-$\beta$2 (Kohase et al., 1986)] in a variety of cell types. These latter observations suggest that TNF-$\alpha$ may also be involved in the control of cell proliferation and differentiation.

Several experimental data suggest that TNF-$\alpha$ may play a role in thymic lymphopoiesis. It stimulates thymocyte proliferation *in vitro* in association with other cytokines: It synergizes with IL-2 and IL-7 on the CD4-CD8- double-negative subset of adult thymocytes (Suda et al., 1990b) and with IL-2 on day-15 murine fetal thymocytes (Suda et al., 1990a). It induces the production of IL-6 by fetal thymocytes (Ranges et al., 1988) and the expression of IL-2 receptors on their cell membrane (Lowenthal et al., 1989). Moreover, TNF-$\alpha$ stimulates the capacity of some thymic epithelial cells to establish interactions with immature thymocytes (Defresne et al., 1990).

The mechanisms by which TNF-$\alpha$ exerts its activities are still unknown, as well as the nature, frequency, and ontogeny of the producing cells in the thymus. All the data available on its production come from *in vitro* studies with thymocytes stimulated by PHA (Ranges et al., 1988), PMA and calcium ionophore (Suda et al., 1990a), or ConA and TPA (Reem et al., 1989). These *in vitro* data do not yield data on the events inside the intact organ. Therefore, we opted for using *in situ* RNA–RNA hybridization on thymus frozen sections to study the presence of cells expressing TNF-$\alpha$ transcripts in the developing thymus. In an attempt to characterize the thymic-producing cells, we have simultaneously identified the pres-

*Corresponding author.

ence of acid phosphatase, an enzyme characteristic for monocytes and macrophages (Duijvestijn et al., 1983), which are known to produce TNF-$\alpha$ in many organs (Matthews, 1981).

Our observations clearly demonstrate two waves of TNF-$\alpha$ mRNA production during thymic ontogeny: The first one occurs at day-14 of gestation; the second one starts at day 16 and reaches its highest level around birth. The nature of the cells responsible for these two waves is not yet identified. Because only a small fraction of thymic cells is expressing TNF-$\alpha$, even at the peak level of production at day 20, and because they are located in the thymic cortex, it could be suggested that thymic nonlymphoid cells (e.g., nurse cells) participate in this production.

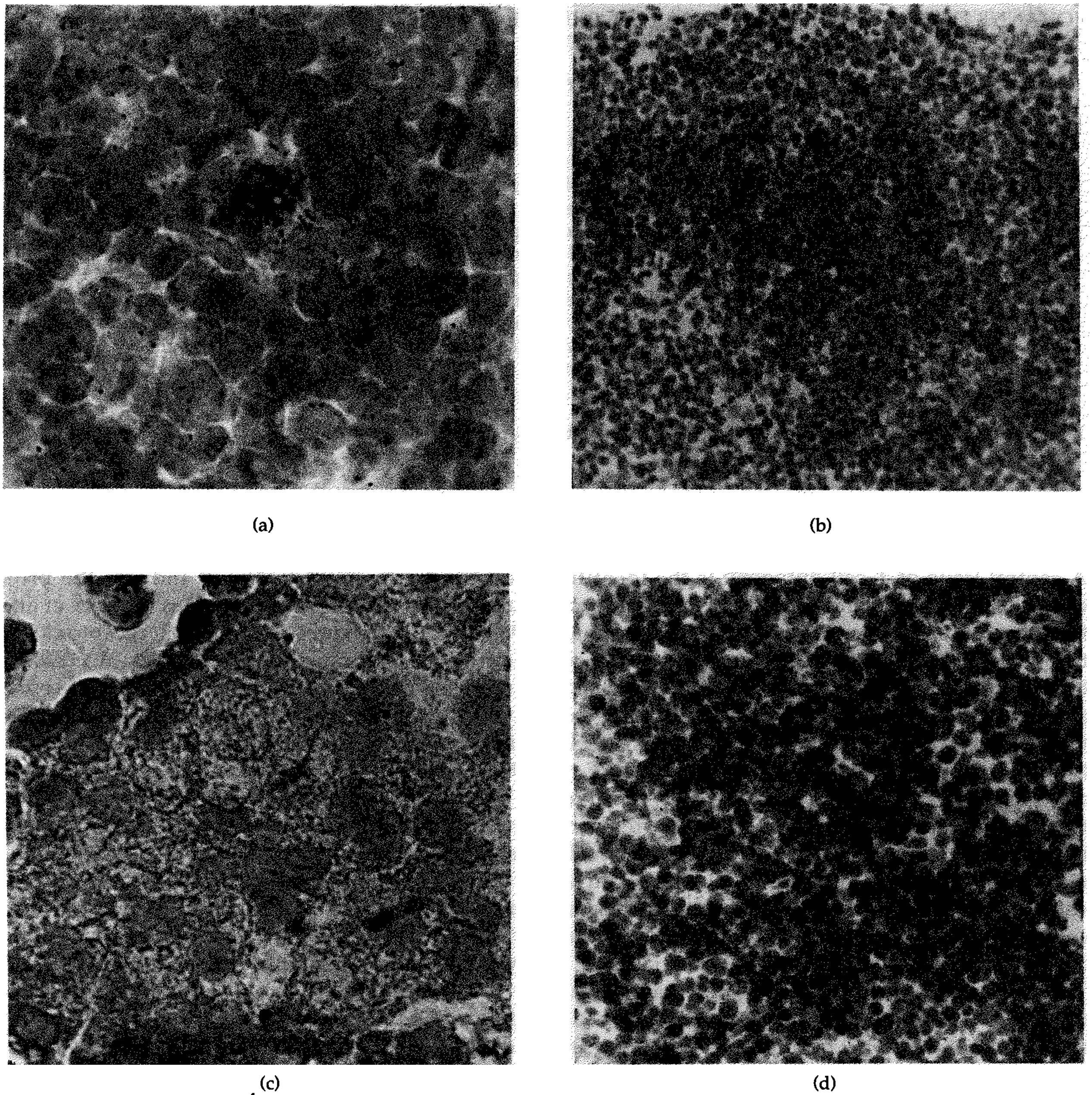

FIGURE 1. Detection by *in situ* hybridization of TNF-$\alpha$ mRNA in fetal thymus frozen sections. Cells containing TNF-$\alpha$ mRNA are identified by the presence of clusters of dark grains (A), they are located in the cortex, C, at day 20 of gestation (B). No hybridization is seen with the control sense probe for TNF-$\alpha$ (C) or after pretreatment of the sections with RNAse (D). (A, C: ×5400; B,D: ×1350).

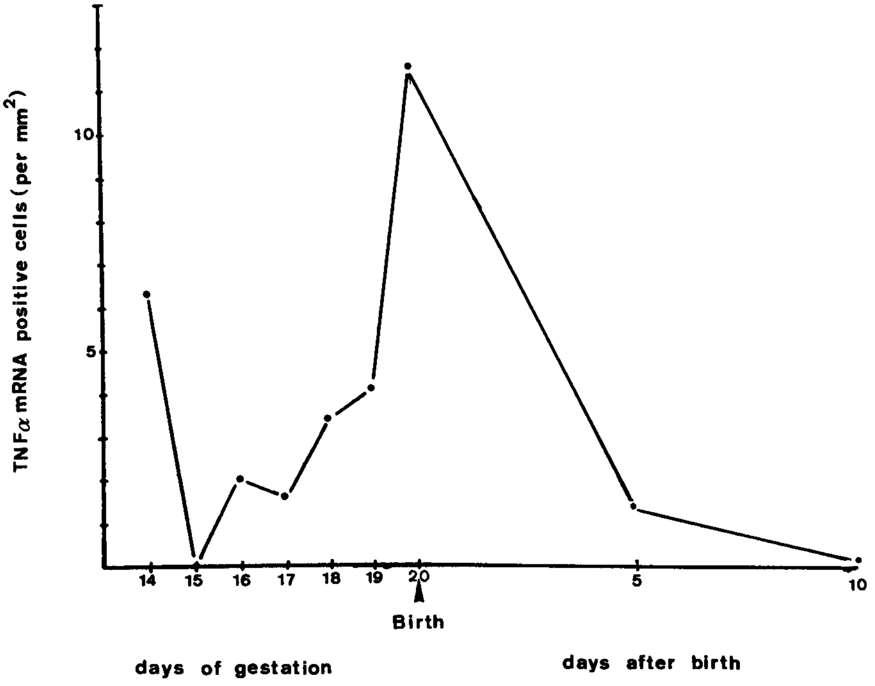

FIGURE 2. Frequency of thymic cells producing TNF-α mRNA during ontogeny as assessed by RNA-RNA *in situ* hybridization: The total number of positive cells was counted on the totality of each section using a light microscope (Leitz) and the section area was estimated with an image analyzer (IBAS).

### RESULTS

### Production of TNF-α mRNA in the Thymus During Ontogeny

As shown in Fig. 1, cells that produce TNF-α mRNA were detected on frozen sections of fetal thymuses (Fig. 1A). These cells were located in the outer part of the thymic lobes and in the cortex at day 20 of gestation when the cortex and the medulla were clearly segregated (Fig. 1B).

There was a striking age-dependent distribution of TNF-α mRNA-producing cells (Fig. 2). Within 14-day thymuses, a significant number of cells contained TNF-α mRNA. A second phase of cytokine production started in 16-day thymuses: the density of positive cells increased progressively up to 20 days of gestation. In between these two waves of lymphokine production, 15-day fetal thymic cells did not express any detectable TNF-α mRNA. Similarly, the density of cells showing TNF-α gene expression decreased immediately after birth.

The specificity of hybridization was assessed using a TNF-α probe of the sense orientation (i.e., the same polarity as cellular RNA) or by pretreating the sections with RNAse. Under such control conditions, no detectable hybridization signals were seen (Figs 1C and 1D). This chronology of TNF-α mRNA production in the fetal thymus was reproduced in five independent experiments.

### Acid Phosphatase Activity in the Thymus During Ontogeny

Because cells of the histiocytic lineage are the most frequent TNF-α producers in a variety of tissues or organs (Matthews, 1981), we identified macrophages in fetal thymuses by histoenzymology (Duijvestijn et al., 1983).

Acid phosphatase-positive cells were not observed within 14-day fetal thymuses (Fig. 3A and 4). The first positive cells appeared at day 15 and their frequency progressively increased to reach a maximum between the day 20 of gestation and the fifth day after birth. These cells were located both in the cortex and in the medulla (Fig. 3B).

### DISCUSSION

In this work, we explored the TNF-α mRNA-producing capacity of fetal thymuses. We detected two waves of cytokine production: The first one occurs on day 14 of gestation, and the second one

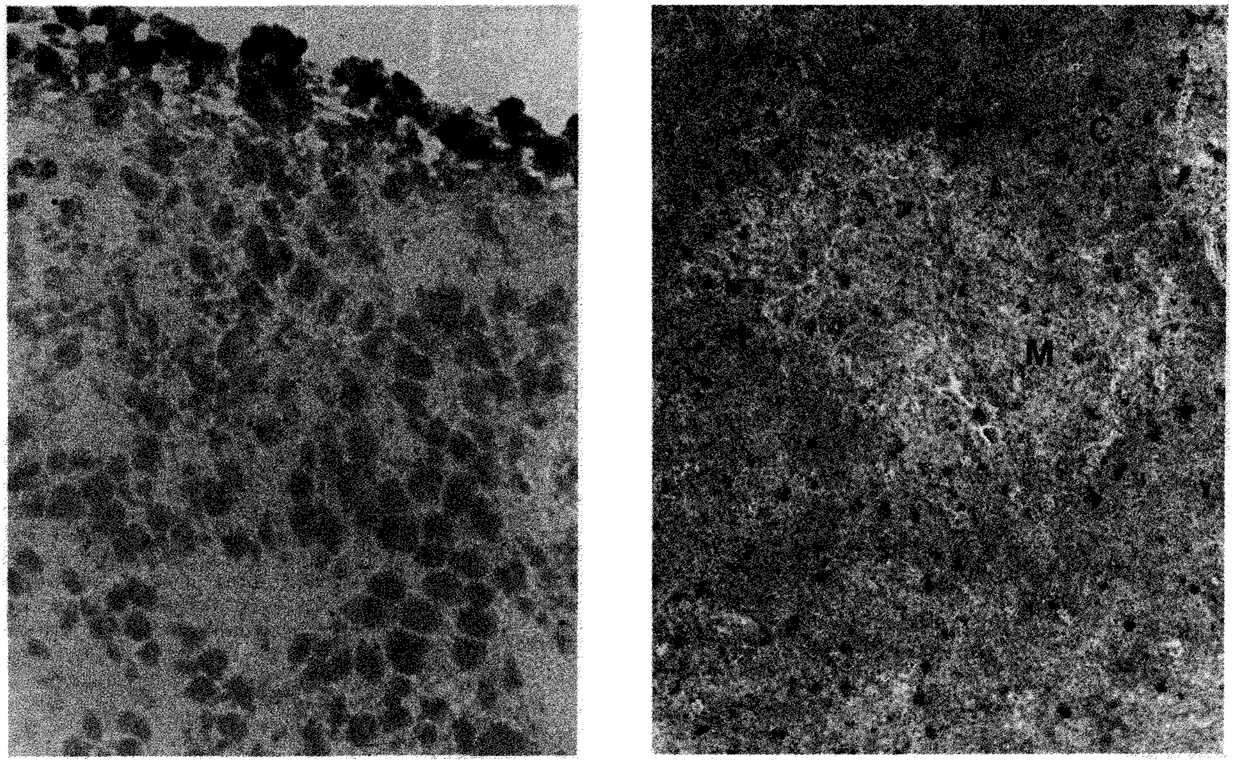

(a) (b)

FIGURE 3. Detection of acid phosphatase-positive cells in fetal thymus frozen sections: They are identified by the presence of granulations within their cytoplasm. No positive cells are observed within 14-day thymuses (A) (×2160). They appear at day 15 and their frequency increases progressively up to day 20 (B) (×540): they are located in the cortex, C, and in the medulla, M.

peaks on day 20. The specificity of the reaction was assessed by using TNF-$\alpha$ probe of the sense orientation or by pretreating the sections with RNAse. The low density of cytokine mRNA-producing cells may explain why it has not been possible to detect constitutive TNF-$\alpha$ mRNA production in fetal thymuses with conventional Northern blot analysis (data not shown).

In an attempt to identify the cells responsible for this TNF-$\alpha$ production, we looked for acid phosphatase activity, which is a good marker of thymic macrophages (Duijvestijn et al., 1983). Acid phosphatase-positive cells are not observed in 14-day thymuses. This suggests that cells producing TNF-$\alpha$ mRNA at this age of fetal development do not belong to the histiocyte lineage. They might be immature thymocytes: 14-day thymuses are indeed essentially composed by epithelial cells and immature CD4-CD8- double-negative thymocytes (Fowlkes and Pardoll, 1989), which can be induced *in vitro* to produce TNF-$\alpha$ (Suda et al., 1990a). Later on, the frequency of acid phosphatase-positive cells increases up to birth. The highest frequency of TNF-$\alpha$ mRNA-expressing cells and the highest levels of acid phosphatase activity are almost simultaneous in 20-day thymuses. However, it appears that only cortical cells express TNF-$\alpha$ mRNA because the hybridization signal is only observed within the cortex, whereas acid phosphatase-positive cells are located on the whole thymic section. It is thus not clear that there exists a subpopulation of thymic macrophages capable of producing TNF-$\alpha$ together with thymocytes, however, it can be suggested that other stromal cells (e.g., nurse cells) participate in this production.

These observations suggest that TNF-$\alpha$ mRNA production by thymic cells is controlled during the ontogeny: These cells may thus be receptive to the inductive signals required for cytokine production only at distinct stages of their development *in vivo*. This is in agreement with recent observations (Carding et al., 1989) that the ability of fetal thymocytes to produce IL-2 and IL-4

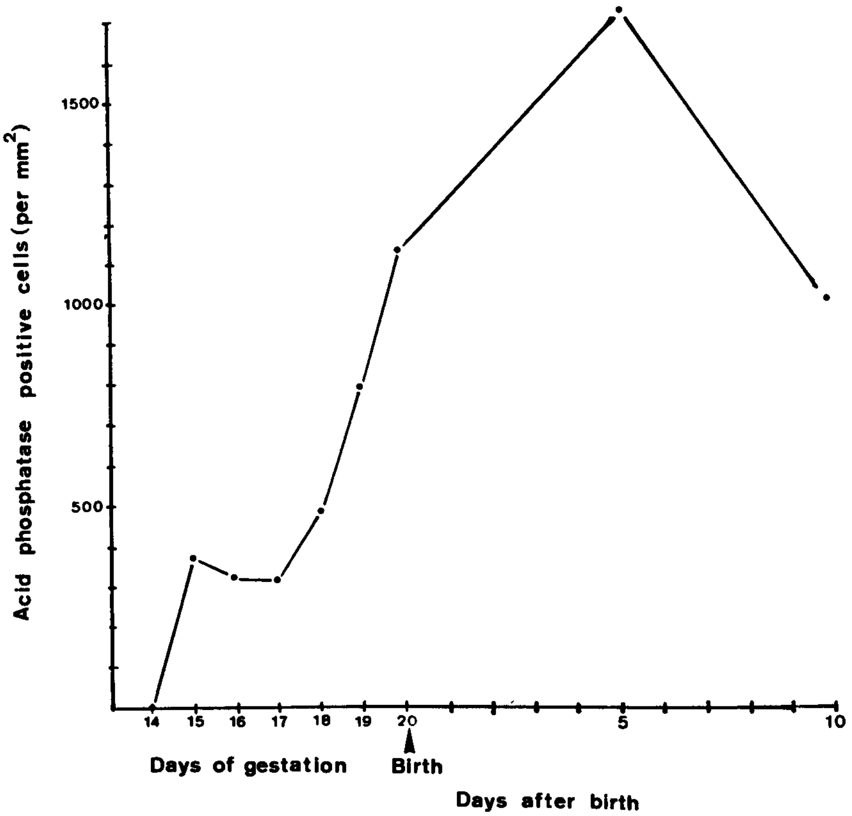

FIGURE 4. Frequency of acid phosphatase-positive cells in the thymus during ontogeny assessed by histoenzymology. The total number of positive cells and the section area are estimated with an iamge analyzer (IBAS).

mRNA as well as IL-2-receptor mRNA appears to be dependent upon the developmental stage of thymocytes.

The signals inducing TNF-α gene expression within the thymus are presently unknown. In vitro studies have shown that CD4-CD8- double-negative adult thymocytes produce TNF-α after stimulation with PMA and calcium ionophore (Suda et al., 1990b). Similarly, human thymocytes treated by PHA produce TNF-α (Ranges et al., 1988) and when stimulated by ConA and TPA, they express TNF-α mRNA (Reem et al., 1989). The physiological equivalents of these activating agents are unknown. It has been shown that IL-1, IL-2, or IL-4 can modulate this production (Ranges et al., 1988); moreover, the peaks of IL-2 and IL-4 mRNA expression in the fetal thymus immediately precede the waves of TNF-α production (Carding et al., 1989). This suggests that these cytokines produced *in vivo* and probably acting in synergy may be required to induce the TNF-α secretion.

This cytokine may be crucial for the proliferation of fetal thymocytes. Whereas it is unable by itself to stimulate the proliferation of thymocytes *in vitro* (Suda et al., 1990b), it enhances the proliferation induced by IL-2 or by IL-7 (Suda et al., 1990b). Moreover, it stimulates thymocytes to produce IL-6 (Ranges et al., 1988), another growth factor that may be required for T-cell development. Finally, in view of our recent observations that TNF-α enhances the capacity of epithelial cells isolated from thymic nurse cells to establish interactions with immature fetal thymocytes (Defresne et al., 1990), it is possible that this cytokine provides the signals for the expression in some thymic stromal cells of molecules facilitating the cell to cell contacts required for the intrathymic selection processes (Marrack et al., 1988).

### MATERIALS AND METHODS

### Mice

C57BL/Ka mice originating from Stanford University were raised in our animal colony. Fetuses were obtained from timed matings; males and females were accoupled for 1 night; the day when they were separated was designated as day 1.

### Tissue Preparations

Cryosections of thymuses (5 to 6 μm) prepared at −20 °C were placed on glass slides coated with 0.1 mg/mL of poly-L-lysine (Sigma). For *in situ* hybridization, the sections were fixed in paraformaldehyde (4% in PBS) containing 20 mM vanadyl ribonucleosides complexes (VRC, Sigma) and 5 mM $MgCl_2$ for 15 min at room temperature. After washing, sections were treated with 0.25% Triton X-100 (Sigma) in PBS containing 20 mM VRC and 5 mM $MgCl_2$ for 10 min at room temperature, dehydrated, and stored at 4 °C in 70% ethanol. For acid-phosphatase detection, sections were fixed in cold acetone (4 °C) for 10 min and stored a few days in a dessicator at room temperature, or longer at −20 °C.

### Preparation of TNF-α probe

The PstI–PstI fragment from the cDNA clone pGEM–1mTNF contains all of the coding sequences for murine TNF-α. This vector was kindly provided by Professor W. Fiers (Gent University, Belgium). Linearized plasmids were used as templates for the *in vitro* synthesis of RNA probes complementary to the cellular TNF-α mRNA (antisense probe). RNA was also transcribed from the opposite direction (sense probe) and used as a negative control. These probes were labeled by random priming using SP6 or T7 RNA polymerases and $^{35}$S-labeled UTP according to the supplier's recommendations (Boehringer Mannheim, Mannheim, FRG). Approximately 1× $10^8$ cpm were incorporated into RNA per μg of RNA template.

### *In Situ* Hybridization

Fifty microliters of probe mixture [50 μL of 50% formamide containing NaCl (0.6 M), Tris-HCl (10 mM), EDTA (1 mM), 1% SDS, DDT (10 mM), yeast t-RNA (0.25 mg/mL) (Boehringer Mannhein, Mannheim, FRG), 1× Denhardt's (50× Denhardt's=1% Ficoll, 1% polyvinylpyrrolidine, 1% BSA), 10% PEG-6000 (polyethylene glycol-6000) and 1 μL of labeled RNA (150.000 cpm/μL)] was loaded on each slide and hybridization was performed at 50 °C overnight in a humid chamber. As a negative control, a few slides were pretreated for 2 hr at 37 °C in a solution containing 20 μg/mL of RNAse to destroy the mRNA or the antisense probe was replaced by the sense probe. Slides were then washed twice for 5 min with PBSM (PBS containing 5 mM $MgCl_2$), rinsed 30 min in a solution containing 20 μg/mL of RNAse (Boehringer Mannheim, Mannheim, FRG) in 0.5 M NaCl and 10 mM Tris-HCl (pH 8) at 37 °C, then 30 min in the same solution except RNAse at 37 °C, 30 min with 50% formamide/2× SSC (2×SSC=0.3 M NaCl/0.03 M sodium citrate) at 50 °C, and 30 min in 50% formamide/1×SSC containing 0.05% of Triton-X at 37 °C. The slides were then dehydrated successively in 30%, 50%, and 70% ethanol in 300 mM ammonium acetate (pH 7.0), air dried, and finally autoradiographed. Ilford K2 emulsion was diluted with an equal volume of 300 mM ammonium acetate. The slides were dipped into the emulsion and allowed to solidify horizontally at room temperature for 4 hr. The emulsion-coated slides were kept at 4 °C for 3–5 days for exposure. They were developed in Kodak D-19 developer (Eastman Kodak, Rochester, NY) for 3 min. After a rinse in a 1% acetic acid solution, the fixation was carried out in an Ilford rapid fixer for 6 min and the slides were washed twice with water for 30 min. They were stained with hematoxilin-eosin for 2 min, washed twice with water, and air dried. We considered as positive cells (cells expressing TNF-α mRNA) those cells that had >8 grains per cell.

### Acid Phosphatases Detection

The substrate used to detect acid phosphatases was the naphtol As-Bi phosphate (Sigma N-2250). The enzymatic reaction was performed as follows: The substrate (60 mg) was dissolved in acetone (3 mL) and 1,2-propyleneglycol (2 mL) and added simultaneously with the coloring Fast Garnet (60 mg) in 60 mL of an acetate buffer (pH 5.0; glacial acetic acid 1 M and sodium acetate 1 M). Fast Garnet is a diazonium salt that coupled and colored the naphtol complexes hydrolyzed by acid phosphatases. The slides were incubated for 30 min at 37 °C in this solution, rinsed with distilled water, and counterstained with hematoxilin for 2 min. Acid phosphatases are lysosomial enzymes and the enzymatic activity in the cells appears as red granulations within the cytoplasm.

### ACKNOWLEDGMENTS

We thank Prof. W. Fiers (University of Gent, Belgium) for the TNF-α probe, Mr. Grosdent (Laboratory of Hematology, Prof. J. M. Paulus, University of Liège) for assistance in techniques of cell count, and Mr. Righetti for iconography. This work was supported in part by the Fund for Medical Scientific Research (Belgium), the Bekales Foundation, the Centre Anticancéreux près l'Université de Liège, and the Fund for Scientific Research of the Faculty of Medecine of Liège.

*(Received May 14, 1991)*

*(Accepted September 25, 1991)*

### REFERENCES

Broxmeyer H., Williams D., Lu L., Cooper S., Anderson S., Beyer G., Hoffman R., and Rubin B. (1986). The suppressive influences of human tumor necrosis factor on bone marrow hematopoietic progenitor cells from normal donors and patients with leukemia: Synergism of tumor necrosis factor and interferon-Y. J. Immunol. **136:**, 4487–4495.

Carding S.R., Jenkinson E.J., Kingston R., Bottomly K., and Owen J.J.T. (1989). Developmental control of lymphokine gene expression in fetal thymocytes during T-cell ontogeny. Proc. Natl. Acad. Sci. USA **86:** 3342–3345.

Carswell E., Old L., Kassel R., Green S., Fiore N., and Williamson B. (1975). An endotoxin-induced serum factor that causes necrosis of tumors. Proc. Natl. Acad. Sci. USA **72:** 3666–3670.

Defresne M.P., Humblet C., Rongy A.M., Greimers R., and Boniver J. (1990). Effect of interferon-gamma and tumor necrosis factor-alpha on lymphoepithelial interactions within thymic nurse cells. Eur. J. Immunol. **20:** 429–432.

Duijvestijn A.M., Schutte R., Köhler Y.G., Korn C., and Hoefsmit E.C.M. (1983). Characterization of the population of phagocytic cells in thymic cell suspensions. A morphological and cytochemical study. Cell Tissue Res. **231:** 313–324.

Fowlkes B.J., and Pardoll D.M. (1989). Molecular and cellular events of T cell development. Adv. Immunol. **44:** 207-264.

Kohase M., Henriksen-DeStefano D., May L., Vilcek J., and Sehgal P. (1986). Induction of β-interferon by tumor necrosis factor: A homeostatic mechanism in the control of cell proliferation. Cell **45:** 659–666.

Lowenthal J.W., Ballard D.W., Bogerd H., Böhnlein E., and Greene W.C. (1989). Tumor necrosis factor-α activation of the IL-2 receptor-α gene involves the induction of kB-specific DNA binding proteins. J. Immunol. **142:** 3121–3128.

Marrack P., Lo D., Brinster R., Palmiter R., Burkly L., Flavell R.H., and Kappler J. (1988). The effect of thymus environment on T cell development and tolerance. Cell **53:** 627–634.

Matthews N. (1981). Tumor necrosis factor from the rabbit. V. Synthesis *in vitro* by mononuclear phagocytes from various tissues of normal and BCG-injected rabbits. Br. J. Cancer **44:** 418–424.

Munker R., Gasson J., Ogawa M., and Koeffler H.P. (1986). Recombinant human TNF induces production of granulocyte-monocyte colony-stimulating factor. Nature **323:** 79–82.

Nawroth P., Bank T., Handley D., Cassimeris J., Chess L., and Stern D. (1986). Tumor necrosis factor/cachectin interacts with endothelial cell receptors to induce release of interleukin-1. J. Exp. Med. **163:** 1363–1375.

Ranges G.E., Zlotnik A., Espevik T., Dinarello C.A., Cerami A., and Palladino M.A. (1988). Tumor necrosis factor-α /cachectin is a growth factor for thymocytes. Synergistic interactions with other cytokines. J. Exp. Med. **167:** 1472–1478.

Reem G.H., Duggan A., and Schleuning M. (1989). Immunoregulation and production of tumor necrosis factor-α by human thymocytes. Cancer Res. **49:** 3568–3573.

Suda T., Murray R., Fischer M., Yukota T., and Zlotnik A. (1990a). Tumor necrosis factor-α and P40 induce day 15 murine fetal thymocyte proliferation in combination with IL-2. J. Immunol. **144:** 1783–1787.

Suda T., Murray R., Guidos C., and Zlotnik A. (1990b). Growth-promoting activity of IL-1α, IL-6, and tumor necrosis factor-α in combination with IL-2, IL-4, or IL-7 on murine thymocytes. Differential effects on CD4/CD8 subsets and on CD3+/CD3– double-negative thymocytes. J. Immunol. **144:** 3039–3045.

